# Land Application of Treated Sewage Sludge: Community Health and Environmental Justice

**DOI:** 10.1289/ehp.1205470

**Published:** 2013-03-11

**Authors:** Amy Lowman, Mary Anne McDonald, Steve Wing, Naeema Muhammad

**Affiliations:** 1 Department of Epidemiology, University of North Carolina, Chapel Hill, Chapel Hill, North Carolina, USA; 2 Department of Community and Family Medicine, Duke University, Durham, North Carolina, USA; 3 Concerned Citizens of Tillery, Tillery, North Carolina, USA

**Keywords:** biosolids, environmental health, environmental justice, land application, qualitative research, sewage sludge

## Abstract

Background: In the United States, most of the treated sewage sludge (biosolids) is applied to farmland as a soil amendment. Critics suggest that rules regulating sewage sludge treatment and land application may be insufficient to protect public health and the environment. Neighbors of land application sites report illness following land application events.

Objectives: We used qualitative research methods to evaluate health and quality of life near land application sites.

Methods: We conducted in-depth interviews with neighbors of land application sites and used qualitative analytic software and team-based methods to analyze interview transcripts and identify themes.

Results: Thirty-four people in North Carolina, South Carolina, and Virginia responded to interviews. Key themes were health impacts, environmental impacts, and environmental justice. Over half of the respondents attributed physical symptoms to application events. Most noted offensive sludge odors that interfere with daily activities and opportunities to socialize with family and friends. Several questioned the fairness of disposing of urban waste in rural neighborhoods. Although a few respondents were satisfied with the responsiveness of public officials regarding sludge, many reported a lack of public notification about land application in their neighborhoods, as well as difficulty reporting concerns to public officials and influencing decisions about how the practice is conducted where they live.

Conclusions: Community members are key witnesses of land application events and their potential impacts on health, quality of life, and the environment. Meaningful involvement of community members in decision making about land application of sewage sludge will strengthen environmental health protections.

In the United States, municipal wastewater must be treated before it is returned to the environment. Sewage sludge is the solid by-product of wastewater treatment. Most of the sludge created by municipal wastewater treatment plants in the United States undergoes biological, chemical, or thermal treatment and is then applied to farmland as a soil amendment [National Research Council (NRC) 2002]. Treated sewage sludge, also called biosolids, contains nutrients useful as fertilizers as well as heavy metals, toxicants, and pathogens. U.S. Environmental Protection Agency (EPA) regulations require periodic monitoring of certain heavy metals and indicator bacteria in treated sludge, but there is no routine monitoring of other toxicants (NRC 2002; U.S. EPA 1994). Most treated sludge is labeled Class B, which has less stringent requirements for pathogen reduction than Class A sludge; the two classes are the same with respect to other contaminants (NRC 2002). Treated sludge is usually applied to land as a liquid spray or solid cake, creating aerosols and dust particles that can drift downwind from the application sites (Baertsch et al. 2007; Paez-Rubio et al. 2007).

Some scientists suggest the rules regulating sludge treatment and land application are based on outdated science and may be insufficient to protect public health and the environment (Gattie and Lewis 2004; Harrison and McBride 2008; Harrison et al. 1999, 2006; Lewis and Gattie 2002; Lewis et al. 2002; Mathney 2011; Snyder 2008). Monitoring land application, enforcing regulatory rules, and systematic tracking and investigation of public concerns are often limited by staffing shortages and budget constraints at federal, state, and local levels (Harrison and Eaton 2001; Lowman et al. 2011; U.S. EPA 2000, 2002). The U.S. EPA’s Inspector General (U.S. EPA 2000) found that,

while EPA promotes land application, EPA cannot assure the public that current land application practices are protective of human health and the environment.

Some residents living near land application sites associate physical symptoms such as mucous membrane irritation, respiratory and gastrointestinal distress, headaches, and skin rashes with land application of sewage sludge (Harrison and Oakes 2002; Lewis et al. 2002; Lowman et al. 2011; Shields 2002). Residents also report foul odors and interference with their quality of life and beneficial use of their property (Lowman et al. 2011; Shields 2002). Although in 2002 the NRC’s Committee on Toxicants and Pathogens in Biosolids Applied to Land recommended studying human exposure and illness, little research into the experiences of persons living near such sites has been conducted since then (NRC 2002).

This article reports the results of analyses of qualitative interviews conducted with neighbors of sites where sewage sludge is applied to land. Qualitative research is of increasing interest in environmental health science, and has been promoted as a useful tool that can complement traditional exposure assessment and epidemiologic studies (Brown 2003; Moffatt and Pless-Mulloli 2003; Scammell 2010). Little quantitative research has been conducted on the impacts of the land application of treated sewage sludge on neighbors’ health because of a lack of systems for surveillance of reported illness (Keil et al. 2011; Lowman et al. 2011), the episodic nature of most applications, and low population density in rural areas. We use qualitative methods to provide detailed information about people’s perceptions of health and quality of life, including temporal sequences of events that may be difficult to ascertain in traditional cross-sectional epidemiologic research. Furthermore, we use qualitative research to understand local and individual factors that may modify a person’s experience with the land application of sewage sludge and to place these experiences into a broader context of environmental injustice.

## Methods

Community members who reported health impacts and nuisances from land-applied sludge near their homes brought this research topic to our attention. We worked with community-based groups in North Carolina and Virginia to identify and invite eligible individuals to respond to an in-depth, semistructured interview about their experiences living near treated sludge application sites. Some eligible participants contacted us after learning about our research through public documents or word of mouth. Interview respondents often referred the interviewers to other individuals who were willing to talk about living near sludge application sites. This recruitment method is a type of purposive sampling commonly used in qualitative research (Merriam 2009; Patton 2002). Rather than using random samples to generalize findings to populations, purposive sampling selects a sample for its ability to provide insight on a research topic (Ulin et al. 2005). Qualitative findings based on purposive sampling may be transferable or relevant to other populations if key elements of the population and context are similar to those of the original research (Bernard 2010; Patton 2002).

To be eligible for the study, participants needed to be ≥ 18 years of age, live within 1 mile of a permitted sewage sludge land application site, speak English, and be willing to spend 1–2 hr responding to a semistructured, open-ended interview about their experiences living near the site. To show appreciation for interviewees’ time, we sent each participant a $25 honorarium.

We (all of the authors) had interviewing experience and all of us conducted interviews between 2009 and 2011. We typically interviewed in pairs at residents’ homes or at private meeting places of their choosing. We completed part of one interview by phone. Often we interviewed two people together, such as a husband and wife. At the beginning of each interview, we explained the research project and obtained informed signed consent from participants to participate in a recorded interview. Interviewers followed a semistructured open-ended discussion guide that included the following topics: participants’ history with the community and their land and what these mean to them; common indoor and outdoor activities; observations or concerns about the surrounding natural environment; perceptions of and experiences with sludge application near their home; individual and community response to the application of sludge; coping mechanisms; and efforts to obtain information, contact authorities, and investigate avenues for action. The guide drew from input from persons living near sludge application sites and from a guide developed for previous research on air pollution from industrial hog operations in North Carolina (Tajik et al. 2008; Wing et al. 2008).

Interviews lasted from 45 min to 2 hr. At each interview, participants provided information about their date of birth, sex, race, and ethnicity. After the interview, researchers wrote or dictated field notes that included observations of the interview context and other information not captured in the recording; for example, descriptions of participants’ homes and yards that provided information on social and economic background, participants’ inaudible reactions that captured depth of feeling on a subject, and observed similarities and differences among participant responses that contributed to the development of themes. When we determined that all interview topics had reached data saturation, that is, when nothing new or contradictory was emerging from the interviews, we concluded data collection.

We transcribed recorded interviews and field notes and reviewed them for accuracy. We read and discussed the interviews as they were completed and transcribed so that early interviews informed later ones. This iterative process enabled us to identify important themes, note areas where we needed more information, and determine whether there were topics that needed further clarification and additional research (Gibson and Brown 2009; Guest et al. 2012; Patton 2002).

As a team, we developed a detailed codebook to analyze the interviews. One team member (A.L.) used Atlas.ti (Scientific Software Development GmbH, Berlin, Germany) to code segments of text from the the interview transcripts and to combine similarly coded passages across all interviews. These grouped passages enabled us to conduct team-based evaluations of the coding, refine code definitions, examine topics that included a range of experiences or opinions, and identify themes and representative quotations (Guest et al. 2012; Ulin et al. 2005). We based our analytic themes and codes on our interview questions and on dominant themes present in participants’ responses. Throughout the analysis we referred to the texts to check that our interpretations were consistent with the data (Guest et al. 2012).

To further enhance the trustworthiness of our analysis, we solicited feedback from five randomly selected respondents about the three main themes we used to summarize our findings and our interpretations of their personal statements. We telephoned the five respondents and presented them with the themes and transcriptions of their interview statements related to those themes. Then we asked whether the themes accurately captured what they said. All five respondents were in complete agreement with the themes and interpretations reported in this article.

The research was approved by the institutional review boards (IRBs) at The University of North Carolina at Chapel Hill (08-0813) and The Duke University Medical Center (Pro00016294). In addition to following the IRB-approved protocol for protecting the confidentiality of study participants, we obtained a certificate of confidentiality from the National Institutes of Health to help protect personally identifiable information from being released in any federal, state, or local legal proceedings, even under court order or subpoena.

## Results

We completed 26 interviews with 34 individuals 35–83 years of age living in rural and semirural areas within approximately 1 mile of sewage sludge land application sites in North Carolina, South Carolina, and Virginia. Twenty participants were from North Carolina, 6 from South Carolina, and 8 from Virginia. Nineteen interviews were with individuals, 5 with married couples, 1 with a brother and sister, and 1 with a married couple and a relative. Of the respondents, 17 were male, 17 female, 21 white, 12 African American, and 1 Hispanic. Interviewers observed that most participants lived in modest homes and neighborhoods that could be described as working or middle class, although a few lived in larger, newer homes that could be described as upper-middle class.

At the time of the interviews, all but 5 respondents had lived in their homes for 5 years or more. Almost half (16/34) of the respondents had lived in their homes or neighborhoods most of their lives, and 11 lived on property or in neighborhoods where their families had lived for more than a generation. Eleven reported having a background in farming. About half maintained gardens on their property, and many tended outdoor animals, including horses, goats, fowl, and dogs.

The study results are categorized according to key themes identified in the interviews about the experience of living near land-applied sewage sludge fields: health impacts, environmental impacts, and environmental justice.

*Health impacts.* Most respondents felt that sludge applications had a negative impact on some aspect of their health. The World Health Organization (WHO) defines health as a state of well-being, and not just the absence of disease (WHO 1948). We drew on this definition to categorize respondents’ remarks on health impacts into the following subthemes: physical well-being, mental well-being, and social well-being.

Physical well-being. Nearly all respondents (30/34) described offensive odors associated with sludge. The extent to which the odor affected the respondents varied. Some described it as “unbearable,” others as an odor they “got used to,” and one respondent said, “it don’t bother me.” Respondents reported they notice sludge odor for periods lasting from 2 days to 6 months after application.

Over half (18/34) of the interview respondents associated acute physical symptoms that lasted a short period of time with sludge application events near their home (Table 1). The most commonly reported symptoms were eye, nose, and throat irritations and gastrointestinal symptoms (nausea, vomiting, diarrhea). Other symptoms reported by more than one respondent include cough, difficulty breathing, sinus congestion or drainage, and skin infections or sores.

**Table 1:** Acute (short duration) physical symptoms respondents attributed to sludge exposure (*n* = 18/34 respondents).

Acute symptom	No. of respondents reporting symptom
Eye, nose, throat irritation	8
Nausea, vomiting, diarrhea	8
Cough	5
Difficulty breathing	4
Sinus congestion, drainage	4
Skin infection, irritation, sore	2

One respondent described recurring physical reactions coincident with sludge applications near her home:

All I know is [the sludge] will make your eyes burn. It will make your throat burn. And then you’ll start coughing, and after that, you can’t breathe. And that’s when I go to the doctors.

A farmer and long-time resident described the nauseating effects of sludge odor:

The stench—it would actually make you sick. It takes a lot to bother me, but it certainly got to me. I’d get nauseated after being out for about an hour in the morning.

Other physical symptoms or conditions that were mentioned by no more than one respondent include pneumonia, swelling of brain arteries, increased seizures, temporary blindness, swollen tongue, closed throat, lung infection, and migraine.

A few respondents expressed concern that they or their family members have chronic health problems, such as asthma or cancer, that make them more sensitive to harmful constituents in sludge. The parents of a child with chronic respiratory problems said they keep him indoors as long as sludge odors from a neighboring field are present—up to 2 or 3 months—to protect him from possible airborne pollutants.

Mental well-being. Over half of the respondents (18/34) said sludge application in their neighborhoods stirred unsettling emotions, including anger, frustration, misery, fear, worry, anxiety, insecurity, and helplessness. Respondents most commonly expressed anger related to not being informed about sludge application in their neighborhood, reckless sludge truck drivers, regulators who seem unconcerned with violations of land application rules, public officials who do not respond to reported concerns, and health impacts.

A woman who reported that she and other family members get sick after nearby sludge applications described the emotional impact of sludge this way:

I’m bitter and frustrated and angry because [sludge] is affecting my family …. And it’s going to alter the rest of their lives because of something that’s been laid down next to them that we knew nothing about, and had no control over.

Malodor from sludge seemed to affect some respondents’ mental states. As one interviewee said,

I’m outside cutting grass or working in the garden and constantly smelling that [sludge] …. Your attitude changes by disturbances in your environment.

A war veteran with posttraumatic stress disorder reported experiencing flashbacks from sludge odor reminiscent of the smell of burning waste in a warzone:

[Sludge] is not just a nuisance; it’s a medical problem for me …. I am not able to get myself to a place where I can begin to heal if they’re constantly driving me backwards … every time I’ve got to walk out of my house and smell the freaking warzone.

Most respondents (26/34) shared ways that sludge odor and other related nuisances interfere with their enjoyment of home, property, and the outdoors. One long-time rural resident who joined her husband in the country after they married volunteered this common sentiment about the impact of sludge odor on her home life:

I don’t want to come home because when we come home, we’re locked in the house. My husband says, “This is not the same. It’s just not the same. We can’t really enjoy where we live.”

Social well-being. Some respondents (8/34) said sludge odors disrupt their opportunities to socialize with family and friends. Several lamented they are unable to spend time walking, playing, eating, or sitting outside as a family when sludge odor is present. One father said,

We have a gazebo outside. We sit outside. At least, that was our conversation in planning it. Family-ness. And [sludge] took that away.

A few respondents said they refuse visits from extended family members because of the intensity of the sludge odor and concerns about its health impacts. A mother and grandmother said,

My daughter wants to come up with the grandkids, with the family—I won’t let her come when they’re sludging. She got so hurt one year. “Mommy, we’re coming for a week.” I said, “No, you can’t.”

Others said sludge odors interfere with social gatherings. One respondent whose family has lived in his neighborhood for generations recalled,

They first put [sludge] out right before the Fourth of July …. We had to put our plans to the side on doing something on the outside. We usually have cookouts, but you can’t cook out in nothing like that.

A total of 22 respondents named specific activities they are unable to do because of malodor from sludge during and for up to several months after a sludge application event (Table 2). The most frequently mentioned activity limitations were letting children play outdoors, opening house and car windows, and hosting relatives or outdoor social gatherings. Others include line-drying laundry, walking freely around the neighborhood, gardening or working outside, sitting outside as a family, and staying home. A few respondents described ways of coping with the odor so they could continue their usual activities. One woman said she wears a mask to do barn chores when sludge odor is strong. Another said she wears a mask to leave the house when the odor is present.

**Table 2:** Activities respondents said they are unable to do because of malodor from sludge during and for up to several months after a sludge application event (*n* = 22/34 respondents).

Activity	No. of respondents reporting activity limitation
Let children play outdoors	8
Open house/car windows	8
Host relatives or outdoor social gatherings	6
Line-dry laundry	5
Walk freely around the neighborhood	5
Garden or work outside	4
Sit outside as a family	3
Stay home	3

*Environmental impacts.* Over half of the interview respondents (18/34) reported observing land application activities of environmental concern to them. The most commonly reported concerns include sludge spillage on public roadways and private property, grazing cattle on land-applied pasture soon after application, the absence of signage at land application sites, and sludge runoff into surface waters. Table 3 lists these and other observations of concern to respondents, as well as the number of respondents who reported them. In some cases, self-informed respondents said that the land application activities they observed were violations of state standards and that they attempted to report them to officials. In other cases, respondents had no knowledge of their state’s land application standards.

**Table 3:** Number of respondents reporting observations of environmental concern (*n* = 18/34 respondents) regarding land application operations

Reported observation	No. of respondents reporting observation
Sludge spillage on road, path, or property	9
Cattle grazing < 30 days after an application event	7
No signage marking application sites during and after application events	6
Sludge runoff into surface waters	5
Sludge in buffer zones (e.g., across property lines, near ditches, gardens, and private wells)	4
Failure of sludge to assimilate into soil	3
Unmarked application boundaries	2
Application during rain event	2
Application in critical watershed	1

About one-third of the respondents (12/34) said they noticed changes in the natural environment since sludge application began in their neighborhood. For example, seven respondents said they noticed more deaths and illness among livestock and water life:

I look at the sludge on this slope—when they put it out, if it rains, this water flows down in this branch …. Now there is no fish or anything that lives in these little branches. No crawdads, anything …. When I was growing up, we’d go there and I would fish for them and so forth. But all this is gone …. So that is saying something has killed all this stuff.

Five respondents reported a change in private well water since applications began near their homes, such as the presence of chemicals, “green slime,” bacteria, or odor. One report came from a man whose property is adjacent to a land application site:

My well … water had an awful smell to it, and a green slime … like three months [after sludge application] …. Before they [applied sludge], I had lived here … two and a half years. Without a problem.

*Environmental justice.* The U.S. EPA (2012) defines environmental justice as the “fair treatment and meaningful involvement of all people … with respect to the development, implementation, and enforcement of environmental laws, regulations, and policies.” Seventeen of 34 respondents indicated they live near sludge application fields that are owned by individuals or entities, including municipalities, who do not live in the community. In light of this, some said their rural or semirural community was being used unfairly as a “dumping ground” for city waste and that they were left to deal with the odor, health problems, and other nuisances that come with it. Four respondents suggested they may be treated inequitably when sites are selected for land application because of their rural and lower income status:

They’ve just got to have somewhere to dump the stuff, and the rural communities, where you’ve got low income people who aren’t able to fight for themselves and stuff like that. That could be some of it.

Related to the “meaningful involvement” component of environmental justice, most respondents described barriers to obtaining information about sludge application in their neighborhood, reporting concerns and problems to public officials, and influencing decisions about the use of sludge where they live. We used these three aspects of “meaningful involvement” to categorize what respondents said on the topic into three subthemes: public notification, reporting concerns, and influencing decisions.

Public notification. All respondents told us that neither public officials nor land appliers directly informed them that sewage sludge from wastewater treatment plants would be applied near their homes. Nearly all expressed disappointment about this. One respondent who reported sludge odors that smelled like “death” and blamed sludge for contaminating his well water described resentment that nobody informed him that a neighboring city would apply sewage sludge a few hundred feet from his home:

We have no knowledge about this, so therefore we’re not prepared for the surprises that may come …. If somebody wants to come out here and explain something to us and it sounds common sense and legit, we’ll listen. Don’t do us like you’re doing us now.

A few respondents mentioned that some municipalities or land appliers post signs to inform the public that land application is occurring but that it is not an effective form of notification because the signs are often difficult to see and interpret. One respondent described a “crumpled up and rusty sign down on the ground.” He said new signs have since been posted but they are not posted at every “sludge field.” Another respondent said she saw a sign by a field in the early days of land application near her home, but at the time she did not understand the terms on the sign, such as “biosolids*,” “*residuals*,”* and “Nutriblend,” which she interpreted to mean they were “applying vitamins.” Others noted that signs were too small or in obscure places, listed incorrect or no contact information, were not posted far enough in advance of application for residents to be prepared, or were present for only a few days rather than the entire application period, which made them easy to miss. Six respondents volunteered that they had not seen signs marking fields where land application was occurring.

Lacking information about land application of sewage sludge, interviewees spoke about their efforts to find out about it. Some said they discussed it with neighbors. At least seven made calls to public officials. Three of the seven said they received straightforward answers about land application of sewage sludge from public officials. Four described difficulty reaching officials and receiving satisfactory answers. For example, they described being transferred on the telephone multiple times and never reaching anyone who would give them straight answers. They said officials responded to their inquiries about sludge with ambiguous statements, such as “it’s safe,” “it’s a farming experiment,” “it’s a special fertilizer,” or “it’s approved.” One woman said that she and her neighbors did not learn the truth about what was being applied in their neighborhood for several years after she first asked a local wastewater treatment official about it. Residents of a different neighborhood reported that when public officials evaded their questions about sludge, they resorted to following sludge trucks to find out what they were hauling.

Reporting concerns. Fourteen respondents said they reported specific sludge-related concerns to officials, including offensive odors, land application in the rain, sludge run-off into drinking water sources, land application in critical watersheds, sludge that fails to assimilate in the soil, suspected well water contamination, reckless sludge trucks, health problems concurrent with sludge application, sensitivity of children and elderly to sludge due to respiratory infections and an immunocompromised condition, inaccuracies in state land application records, and questions about the heavy metals content or general safety of the sludge. A few respondents reported improvements in the land application practice over time and said officials and operators had responded to their concerns by respecting set-back distances, using alternate driving routes, slowing down trucks hauling sludge, posting correct contact information on land application signs, and returning their phone calls requesting information.

Nearly all (13/14) respondents who reported concerns registered dissatisfaction overall with the response from officials, saying they “do nothing,” “don’t listen to the people,” answer to the industry rather than the people, “beat around the bush,” “sidestep stuff,” “deny there’s a problem,” “don’t investigate concerns,” “don’t keep their word,” don’t answer their phones, try to cover things up, say contradictory things about the constituents of sludge, act “like they don’t care,” and have no interest in doctors’ letters stating it is unsafe for their patient to be exposed to sludge.

Influencing decisions. One respondent described feeling “powerless” to influence land application in his community because all the power and control are with the sludge industry, and local leadership will not or cannot do anything to change the practice. Similar frustration was expressed in other interviews. For example, a respondent from Virginia said the Dillon Rule, a judicial doctrine that limits local government authority in Virginia, North Carolina, and other states (Clay 1989), prevents her local government from establishing rules and regulations governing land application where she lives. She felt that it was unfair to favor one land owner who wants to use sludge when the majority of the community is opposed to it. She added,

The industry has all the control. Because they can pull up application, or they can lay it down. And they don’t care. As long as they’ve got permission to do it, they’re going to do it.

In spite of perceived barriers to influencing land application decisions, over half of the interviewees (19/34) described changes they would like the industry to make to improve public notification and enhance public and environmental protections. First, several respondents suggested public officials should directly notify residents within 1 mile of sludge fields before the first and subsequent land application events. A few said residents should be given the opportunity before land application events to inform public officials of household members with health conditions, such as a respiratory illness or weakened immune system, so that an injection method of land application can be used to better safeguard their health, or so application at the site can be suspended.

Some respondents who reported poorly visible signs near sludge fields or who reported seeing no signs at all suggested that land appliers post large visible signs 2 weeks before application and for the duration of the event. Respondents said this would allow them to prepare for the event and take necessary safety precautions for their family and animals. Also related to public notifications, some respondents said they would like to receive the results of sludge testing from the wastewater treatment plants that apply waste near their homes in order to monitor concentrations of harmful constituents and possible concerns.

Respondents concerned about well water contamination said the city should provide water to residents in land application areas or offer free periodic testing of their private well water to evaluate its safety. A few respondents said application in a critical watershed and land application before forecasted rain events should be prohibited. If the latter should occur, respondents said the sludge should be tilled under immediately following application to prevent runoff. Some respondents also felt that land application should not occur under windy conditions because of the increased likelihood of exposing neighbors to migrating pollutants. Generally speaking, respondents who were aware of land application rules and who reported violations said that better enforcement of existing rules is needed to protect human and environmental health.

Respondents who felt there were conflicts of interest in land application governance and practice that undermine human health and the environment said these should be minimized by contracting with independent scientists to perform and report soil and sludge batch testing; funding independent, formal research about health and environmental impacts of sludge application; prohibiting state and local health departments and the U.S. EPA from promoting land application; and making government employees responsible for telling residents the truth about land application.

Finally, a few respondents said they would like the land application industry to improve and maintain roads damaged by the frequent travel of heavy sludge trucks.

Overall, eight respondents said they would like land application to stop, either indefinitely or until independent research can “prove it’s safe” for human health and the environment.

## Discussion

We used qualitative research methods to enhance understanding of the impacts of land-applied sewage sludge on the health and quality of life of nearby populations. Respondents reported symptoms consistent with findings from earlier studies that report neighbors of land application sites experience physical reactions to land-applied sludge (Gattie and Lewis 2004; Lewis et al. 2002). Confined animal feeding operations (CAFOs) also apply liquid wastes and sludge to farmland. CAFO neighbors describe health impacts similar to those reported by neighbors of land-applied sewage sludge (Bullers 2005; Horton et al. 2009; Radon et al. 2007; Schiffman 1998; Schiffman et al. 2000; Schinasi et al. 2011; Tajik et al. 2008; Thu 2002; Thu et al. 1997; Wing and Wolf 2000; Wing et al. 2008). The overlap of hazardous agents in CAFO waste and treated sewage sludge, including odorant compounds, endotoxins, and other allergens and irritants, suggests that similar community health impacts are plausible (Lewis et al. 2002).

Respondents also reported adverse impacts on their mental and social well-being and on the surrounding natural environment. Some said they observed sludge spillage on public roadways and private property, grazing cattle on land-applied pasture soon after application, and sludge runoff into surface waters. These and other land application activities that respondents said they witnessed are violations of land application standards in one or more of the three states represented in this study (Harrison and Eaton 2001; North Carolina Department of Environment and Natural Resources 2006; South Carolina Department of Health and Environmental Control 2009; Virginia Department of Environmental Quality 2011), highlighting the need for stricter enforcement of standards.

Respondents also described environmental injustices related to land application of sewage sludge, including barriers to participating in decisions about how the practice is conducted in their neighborhood. Land application of sewage sludge is part of a larger context of environmental injustice that characterizes relationships between urban areas that create wastes and nearby rural areas that receive the wastes. In addition to sewage sludge, urban wastes disposed in rural and semirural communities include municipal solid wastes, construction and demolition debris, and industrial wastes (Norton et al. 2007). Jones (2011) describes the urban–rural dimension of environmental injustice this way:

For the majority of Americans who live in metropolitan areas, rural dumping becomes a logical choice: undeveloped land is inexpensive and available, fewer residents will be harmed should containment measures fail, and, most importantly, nuisances and dangers are removed from their own neighborhoods.

This report does not include everything respondents said about living near sludge application sites; rather it represents the dominant themes that we identified in the open-ended interviews. There were few positive remarks about sludge and the response of industry and government officials to residents’ concerns, possibly because of our method of recruiting participants. We asked community contacts to help us identify people who could provide information on the subject of living near sludge application sites. Although we did not ask for referrals to people who had problems with sludge, people with negative opinions of the practice may share local social networks, which could lead to their perspectives being overrepresented. Alternatively, some rural residents who have been negatively affected by land application of sludge may be unwilling to speak out or participate in research because they fear retribution from influential land owners or government officials who benefit from sludge application and control rental property, access to resources, or jobs. In addition, we are unable to report the numbers of respondents who had similar or opposing views or experiences for all interview topics because we obtained the information through open-ended interviews that did not probe the participants to respond to a list of standardized questions. Our study was not designed to quantify the prevalence or incidence of reported symptoms, health impacts and other concerns in populations near land application sites.

Our study does demonstrate that people of diverse backgrounds who live in three different states raised health and environmental concerns about land application. Similarities in participant statements, issues raised, and terminology used suggest that the health and environmental issues identified here warrant attention from environmental health scientists and public health officials. Although differences in the composition and treatment of sewage sludge, land application methods, and geographic features of application sites make the transferability of results to other locations uncertain, case reports indicate that similar health and quality of life issues are raised in other states and countries (Harrison and Oakes 2002; Lewis et al. 2002; Lowman et al. 2011; Shields 2002).

## Conclusion

Most respondents suggested that if land application continues, it should be conducted in a more just and democratic way—one that informs people who may be affected by the application before it occurs, takes community input seriously and adapts the practice accordingly, and ensures that people and their environment are kept safe from harm.

Phil Brown (2003), a professor of sociology at Brown University who has studied contaminated communities worldwide, concluded,

Virtually all cases of contaminated communities are detected by lay discovery, largely because affected populations tend to notice environmental problems. As well, scientists and government agencies are not usually carrying out routine surveillance that would detect such problems.

Surveillance and monitoring of land application of sewage sludge is limited, and enforcement of the rules is weak (Harrison and Eaton 2001; Lowman et al. 2011; U.S. EPA 2000, 2002). Community members are key witnesses of land application events and their potential impacts on health, quality of life, and the environment. As such, they may consider documenting their experiences by taking photographs and keeping diaries with dates, times, and descriptions of application events, truck traffic, odor, physical reactions, environmental impacts, or other observations. Residents’ documentation and ideas for improvements to land application offer a distinct perspective on the practice that industry and government officials lack. Meaningful involvement of community members in decision making about land application of sewage sludge will strengthen environmental health protections.
